# Ham Yeasts: Exploring Mycoprotein Potential Production of Yeasts Isolated from Spanish Dry-Cured Ham

**DOI:** 10.1021/acsfoodscitech.5c00671

**Published:** 2025-09-26

**Authors:** Noelia Viveros-Lizondo, Beatriz García-Béjar, Elena Coso-Cuevas, Almudena Soriano, María Arévalo-Villena

**Affiliations:** † Department of Analytical Chemistry and Food Technology, Faculty of Chemical Sciences and Technologies, 16733 University of Castilla-La Mancha, Camilo José Cela Avenue 10, 13071 Ciudad Real, Spain; § Regional Institute for Applied Scientific Research (IRICA), University of Castilla-La Mancha, Camilo José Cela Avenue 10, 13071 Ciudad Real, Spain

**Keywords:** Spanish ham, ripening rooms, yeast biodiversity, mycoproteins

## Abstract

Ripening conditions of dry-cured ham provide a suitable environment for microbial growth. Although salts such as sodium chloride, nitrite, and nitrate act as inhibitory agents, certain microorganisms, particularly yeasts, can still develop. This study evaluates the biodiversity and biotechnological traits of yeasts isolated from Spanish dry-cured hams, comparing natural and controlled drying rooms and assessing their potential for protein production. Samples were collected from five drying rooms, including both Serrano and Iberian hams as well as ham hangers and air. The average yeast population was 5.44 ± 1.29 log CFU/cm^2^, with no growth detected in air samples. Non-*Saccharomyces* species were more prevalent than *Saccharomyces*, with *Debaryomyces hansenii* and *Yarrowia lipolytica* being the most dominant due to their strong proteolytic activity, which contributes to ham flavor and texture. Natural drying rooms exhibited greater yeast diversity and higher counts. Selected yeast strains were evaluated for their potential as mycoprotein sources through kinetic and protein production analyses. *Debaryomyces hansenii* showed the highest protein content (495.11 ± 22.19 mg/g dry weight), making it a promising candidate for low-animal-protein meat alternatives.

## Graphical abstract

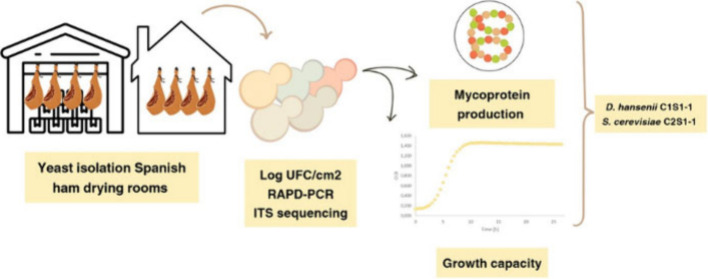

## 1 Introduction

Two main types of Spanish dry-cured ham can be distinguished: Iberian ham, made from pigs of pure Iberian breed or from crossbreeds of Duroc males and Iberian sows that contain a maximum of 50% Duroc blood; and white dry-cured ham, as Serrano ham, produced from white pig crossbreeds including Landrace, Large White, Duroc and Pietrain.

The production of cured ham aims to both achieve microbiological stabilization of the final product and enhance its sensory qualities. Factors such as the pig breed, animal diet, processing conditions and ripening time must be considered since, as they are crucial for the final characteristics of the product.^1^ Ripening is a critical stage, influencing the ham’s final characteristics through gradual dehydration. This process may occur in industrial drying rooms with controlled conditions (e.g., temperature, humidity, and air recirculation), enabling faster, large-scale, and uniform production.^2^ Alternatively, natural drying, typically used for Iberian ham production, relies on specific climate conditions that are valued for its artisanal quality. However, this traditional method is slower, seasonal, and may produce variable results between batches.^3^

During the ripening stage, several biochemical reactions occur, generating numerous low-molecular-weight compounds and volatile compounds that influence the final quality of the product.^4,5^ Most of these reactions are mediated by endogenous enzymes, such as proteases and lipases, as well as by proteases released by naturally present microorganisms.

The predominant microorganisms are micrococci, yeasts and molds, with lactic acid bacteria (LAB) present to a lesser extent.^6^ It is essential to monitor and control the microbial growth at the different production stages, as it directly affects the final characteristics of the ham. Yeasts and molds show high resistance to the low water activity typical of the ripening process, whereas bacteria are more sensitive to this parameter: micrococci population drops at the end of curing stage, while LAB gradually decreases during drying. The presence of microorganisms during processing contributes to the desirable aroma, flavor and texture of the product.^7,8^

The presence of different microorganisms is closely related with the geographical area of ripening and the type of drying room.^9,10^ Yeasts have a clear influence on the typical sensory characteristics of the dry-cured ham, with specific strains being associated with the production of distinctive volatile compounds, highlighting *Debaryomyces* and *Candida* as the main genera isolated in this product.^8^ Certain secreted fungal proteases and lipases during the ripening process have a direct impact on the development of volatile compounds that determine odor and flavor, as well as on the texture of the ham.^11^ To a lesser extent, nonmicrobial reactions, such as the Maillard and the Strecker reactions, also contribute to formation of volatile compounds, which typically occurs under conditions of heat or prolonged storage.^11,12^ In fact, Martín et al.^13^ highlighted the relevance of microorganisms in ham production, noting that the typical flavor of cured products results from the combined effects of enzymatic action and microbial growth.

Due to their metabolic versatility, rapid growth, and ease of genetic manipulation, yeasts are excellent candidates for the production of compounds of industrial and biotechnological interest. Their applications span multiple sectors, including food production (e.g., fermentation of beverages), the pharmaceutical industry (e.g., synthesis of biopharmaceuticals and secondary metabolites) and bioremediation (e.g., degradation of environmental pollutants).^14^ In the current context, where food sustainability and reduction of environmental impact are global priorities, there is a pressing need to reduce animal protein consumption and develop more sustainable alternatives. In this regard, yeasts stand out as a promising solution due to their ability to synthesize high-quality proteins and provide essential nutrients, thus contributing to the diversification of protein sources in the food industry.

Therefore, the aim of this work was to explore the mycoprotein production potential of yeast strains isolated from Spanish dry-cured ham related environments. To this end, the study first assessed the biodiversity of yeast population present during the ripening phase in both controlled and natural drying rooms and subsequently characterized the identified strains in terms of kinetic parameters and protein production for a future application on the development of new mixed meat products with a lower protein animal content.

## 2 Materials and Methods

### 2.1 Sample Collection and Count Yeasts

To obtain a representative yeast population, a total of 34 samples were taken directly from the surface of Serrano and Iberian hams as well as from the drying room environment (ham hangers and air). Sampling was carried out in four facilities (Figure 1): two controlled and two natural drying rooms which differed in humidity, temperature and air circulation.

**Figure 1:**
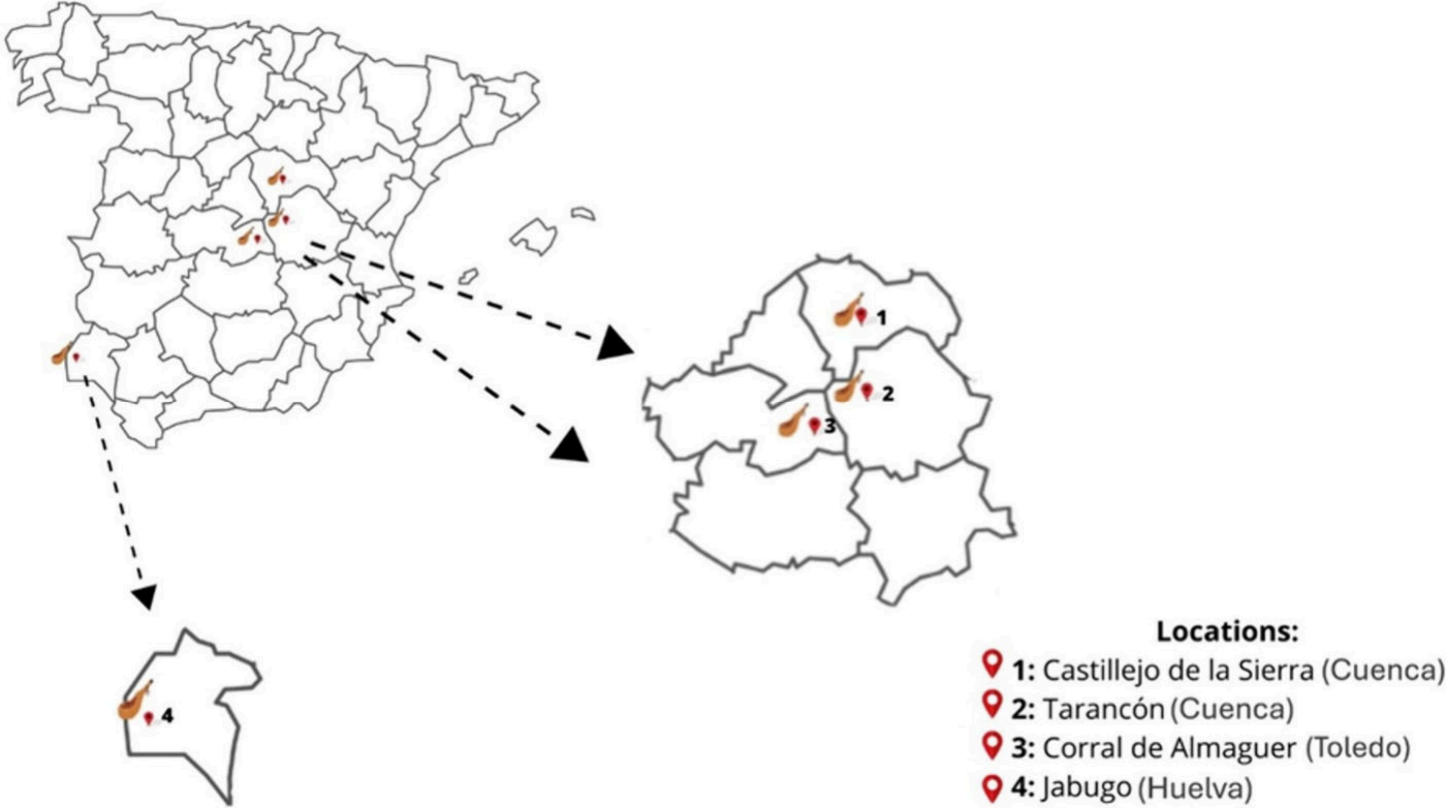
Location of the sample drying rooms.

The controlled drying rooms sampled were located in Corral de Almaguer (Toledo) and Tarancón (Cuenca) and belonged to a leading meat company in Spain (Incarlopsa). Regarding the natural drying rooms, one of them also belonged to this company and were located in Jabugo (Huelva), while the other was located in the Serranía de Cuenca Natural Park (Castillejo-Sierra), belonged to a private individual. In the latter case, samples were taken only from the surface of white and Iberian ham. The sampling points and types of sampling as well as the assigned codes are detailed in Table 1.

**Table 1:** Sampling Points of the Different Cured Ham Drying Rooms^a^

**Controlled drying room**			
**Samples**	**Drying room 1 Corral de Almaguer**	**Drying room 2 Corral de Almaguer**	**Drying room 3 Tarancón**
Serrano ham	C1S1; C1S2; C1S3	C2S1; C2S2; C2S3	C3S1; C3S2; C3S3
Ham hangers	C1H1; C1H2	C2H1; C2H2	C3H1; C3H2
Air	C1A1	C2A1	C3A1

**Natural drying room**		
**Samples**	**Drying room 4 Jabugo**	**Drying room 5 Castillejo – Sierra**
Iberian ham	N4I1; N4I2; N4I3	N5I1
Serrano ham	-	N5S1; N5S2
Ham hangers	N4H1; N4H2	N5H1; N5H2
Air	N4A1	N5A1

a **C** (controlled drying room), **N** (natural drying room), **S** (Serrano ham), **I** (Iberian ham), **H** (ham hangers), **A** (air). A number was assigned to each sampling point to complete the code.

Yeast counts of the cured hams were performed by sampling their surfaces with sterile swabs that were inoculated in YM broth (yeast extract 3 g/L, malt extract 3 g/L, peptone 5 g/L, glucose 10 g/L). Then, 10-fold serial dilutions in NaCl solution (0,9%) were done and were inoculated on the surface of YM agar (yeast extract 3 g/L, malt extract 3 g/L, peptone 5 g/L, glucose 10 g/L, agar 20 g/L) using the Eddy Jet 2 spiral plater (IUL Instruments). In order to avoid bacterial and mold growth, chloramphenicol (100 μg/mL; Panreac, Barcelona, Spain), ampicillin (100 μg/mL; Sigma, Steinheim, Germany), and biphenyl (200 μg/mL; Sigma, Steinheim, Germany) were supplied to both media. All plates were incubated for 48 h at 30 °C, and results were expressed as CFU/cm^2^.

Furthermore, air samples were taken in drying rooms using an air sampler (Spin Air Basic, IUL instruments) with a volumetric flow rate of 2.5 L/min. The air samples were incubated at the same conditions previously mentioned for YM agar, and the results were expressed as CFU/L.

### 2.2 Isolation, Purification and Conservation

From the growth plates, a maximum of 50 isolates with different morphologies were selected from each sample or their dilutions. Then, yeast isolates were streaked on YM agar and incubated at 30 °C for 48 h. After checking the purity of the colonies, all yeast were preserved in 15% glycerol at −80 °C until analysis.

### 2.3 Genotyping, Identification, and Sequencing of Yeasts

To carry out the strain differentiation and subsequent identification, the methodology proposed by García-Béjar et al.^15^ was followed.

Strain pattern determination of yeasts isolated from cured ham environment was carried out using PCR-RAPD (Polymerase Chain Reaction - Random Amplification of Polymorphic DNA). Random DNA fragments were amplified with Taq DNA polymerase (Biotools, Madrid, Spain) and the unspecific M13 primer (5′ GAGG­GGGT­GGCG­GCGG­TTCT 3′) supplied by Condalab (Madrid, Spain). The reactions were performed in a final volume of 20 μL, and amplification was conducted using a Life Touch thermal cycler (Bioer, Barcelona, Spain). The program was as follows: An initial denaturation at 94 °C/4 min, 2 cycles with a denaturation at 94 °C/1 min, a hybridization at 45 °C/1 min and an extension at 72 °C/1 min, 35 cycles consisting of a denaturation at 94 °C/40 s, a hybridization at 52 °C/1 min and an extension at 72 °C/3 min, and, at the end, a final extension at 72 °C/10 min. The amplification products were then separated by electrophoresis on a 2% (w/v) agarose gel and visualized using Gel Green (6×) (Biotium, California City, CA, USA) in a gel documentation system. A DNA molecular weight marker (100 bp ladder, Biotools) was used as a DNA length standard.

After this, all different strains were identified by the PCR-RFLP (Restriction Fragment Length Polymorphisms) technique. The entire internal transcribed spacer (ITS) region surrounding the 5.8S rRNA gene region was amplified with the primer pairs ITS1 (5′-TCCG­TAGG­TGAA­CCTG­CGG-3′) and ITS4 (5′-TCCT­CCGC­TTAT­TGAT­ATA­TGC-3′) (Macrogen, Madrid, Spain) on the Life Touch thermal cycler. Thermal cycling parameters consisted of an initial denaturation at 95 °C for 10 min, followed by 36 cycles of denaturation at 95 °C/1 min, hybridization at 55.5 °C/1 min and extension at 72 °C/90 s, with a final extension at 72 °C/10 min. Then, PCR products were digested with the restriction endonucleases *Hae*III, Hinf I y Cfo II and their corresponding buffers (Thermo Scientific, Vilnius, Lithuania) at 37 °C for 8 h with gentle shaking.^16^ PCR products and their restriction fragments were separated by electrophoresis on agarose gels and visualized following the same protocol as described above. A 100 bp DNA length standard was used for estimating the band sizes. Then, the resulting profiles were identified using the Yeast-ID database (CECT, University of Valencia and CSIC, Spain).

A representative profile of each species was selected for sequencing, and the previously amplified ITS region was sent to Macrogen SPAIN (Madrid, Spain). The received sequences were entered into the bioinformatics tool BLAST (GenBank, Bethesda, MD, USA) in order to corroborate the identification made. The different isolates were included in the culture collection of the Yeast Biotechnology Laboratory (University of Castilla-La Mancha).

### 2.4 Yeast Biodiversity in Cured-Ham Environments

To assess the biodiversity of the studied environments, Simpson index (D) was used, which is defined as a coefficient of species diversity within a given environment or community. This index accounts for the number of species present and the relative abundance of each one among the total number of isolated microorganisms. The resulting value ranges from 0 (no biodiversity) to 1 (infinite biodiversity). This was calculated according to the following equation:

D=(Σn(n−1))/(N(N−1))

where “*n*” is the number of isolates per species and, “*N*” is the total number of isolates per sample.^15^

Moreover, intraspecific genetic variability that exists within the same species was calculated as follows:

Genetic variability (%) = (No. of different strains)/(No. isolates of the same species) × 100

### 2.5 Screening and Yeast Characterization

To select yeasts with the highest mycoprotein production capability, different properties of interest were evaluated.

#### 2.5.1 Growth Capacity

To identify those strains with an adequate growth rate, together with rapid adaptation and high productivity and fast adaptation, the following parameters were evaluated: latency phase (λ), maximum growth rate (μ max), generation time (G) and maximum optical density (OD max).^17^

For this assay, strains were inoculated in 3 mL of YM broth and incubated at 30 °C while being shaken (100 rpm). After 24 h, cultures were transferred to a 96-well microplate (ThermoFisher, Waltham, MA, USA) with 200 μL of fresh YM broth at a final concentration of 5 × 10^6^ CFU/mL. Each experiment was performed by triplicate, and noninoculated YM broth was used as negative control. Monitorization of the growth curve was performed by optical density (DO) measurements at 600 nm every 30 min for 24 h after a brief shaking step, using a Hipo MPP-96 microplate reader (Biosan, Letonia). The resulting OD values were plotted against time (h) to generate graphical representations of the growth curves.^18^

#### 2.5.2 Protein Production

Those yeast strains that meet the criteria established for their kinetic parameters were subjected to another assay where the protein content was evaluated.

To determine the total protein content, cells were disrupted by ultrasound treatment at 50 W for 20 min to release the proteins. These conditions were optimized based on the study Viveros-Lizondo et al.,^19^ where it was demonstrated that there was a greater release of proteins without reaching saturation of the method. After that, protein quantification was carried out using the Bradford method with Coomassie Brilliant Blue reagent.^20^ The dry weight was obtained by drying (60 °C/12 h) 500 μL of wet biomass placed on an aluminum capsule and by calculating the weight difference. The results were expressed as milligrams of protein per gram of yeast dry weight.

#### 2.5.3 Metabolism Inactivation: Growth Inhibition after Ultrasound Treatment

Finally, the strains that exceeded the established protein content threshold were subjected to a viability study after ultrasound treatment. For this purpose, the protocol proposed by Viveros-Lizondo et al.^19^ was followed, after the application of the ultrasound method (50 W/20 min), a 100 μL aliquot of each yeast strain was inoculated on the surface of YM agar plates and incubated for 48–96 h at 30 °C. The assay was carried out in triplicate.

### 2.6 Statistical Analysis

IBM SPSS Statistics 21 was used to perform statistical analyses. A one-way analysis of variance (ANOVA) followed by the Duncan’s test (*p* < 0.05) was applied to study significant differences between kinetic parameter data and protein content after the use of ultrasound.

To determine significant differences between the counts obtained in the natural and controlled drying rooms, the Student’s *t* test was performed (*p* < 0.05). In addition, descriptive statistics were also applied to the kinetic parameter.

## 3 Results and Discusion

### 3.1 Quantification of Yeast Population and Isolation

A higher yeast count was found in Serrano ham ripened in natural drying rooms compared with the controlled drying rooms. Previous studies have reported that natural drying rooms have higher counts, which could be attributed to a lower degree of control of temperature, humidity, and air conditions compared to controlled drying rooms.^10^ However, in other studies, yeast counts are generally higher in Serrano ham samples in controlled drying rooms, which could indicate that they establish conditions for the development of characteristic flavors.^7^

The mean yeast count of the samples was 5.44 ± 1.29 log of CFU/cm^2^. There is great variability in the distribution of yeasts between different sample types and environments (data not shown). In the controlled drying rooms, the Serrano ham samples had a concentration of around 6 log CFU/cm^2^, except for sample C3S1, which had a significantly lower count (2.37 log CFU/cm^2^). The counts of the ham hanger samples were mostly lower, ranging from 2.25 log CFU/cm^2^ (C1H2) to almost 6 log CFU/cm^2^ (C2H2).

More differences were observed between the samples compared to those in the natural drying rooms. Samples N4I1 and N4I3 showed relatively high yeast counts (5.73 and 5.39 log CFU/cm^2^, respectively), indicating a notable presence of yeasts in Iberian ham from natural drying rooms. Samples N4I2 and N5I1 showed an absence of growth, which could be due to the specific conditions of these particular samples. The Serrano ham samples from these drying rooms showed no growth (N5S1); however, sample N5S2 showed the highest count value (6.63 log CFU/cm^2^), suggesting a high concentration of yeast at this particular sampling point.

To compare the Serrano ham counts between the controlled and natural drying rooms, the Student’s *t* test statistical analysis was carried out (Table 2).

**Table 2:** Means ± Standard Deviations of Yeast Counts Log (CFU/cm^2^) in the Controlled Drying Room (C) and Natural Drying Room (N) of Serrano and Iberian Ham, Ham Hangers, and Air^a^

	Serrano ham (S)	Iberian ham (I)	Air (A)	Ham hangers (H)
**Controlled drying room (C)**	5.81 ± 1.26^a^	-	-	4.93 ± 1.37^b^
**Natural drying room (N)**	6.63 ± 0.09^b^	5.55 ± 0.20	-	3.74 ± 0.06^a^

a (−) No yeast growth. Different superscripts in the same column indicate significant differences between the mean values (*p* < 0.05).

Results showed significant differences (*p* = 0.015) between the two drying room types. It was observed that the natural drying room has a higher count compared to the controlled drying room. The variation in yeast counts between controlled and natural drying rooms is evidence of the influence of environmental conditions on the microbiota of Serrano ham. Previous studies have reported that natural drying rooms have higher counts, which could be attributed to a lower degree of control of temperature, humidity, and ventilation conditions compared to controlled drying rooms.^10^

Likewise, the analysis of the hangers showed counts significantly higher in the controlled drying rooms, suggesting that the environmental and operational conditions of the drying process may modify the microbiological dynamics of the product.

No yeast growth was detected in the air samples in any type of drying room, only bacterial and mold growth (data not shown). This could be due to the air control conditions, which suggests a proper management to prevent the proliferation of unwanted yeasts, corroborating the importance of controlled conditions.^10^

The presence of yeasts in products such as dry-cured ham can have positive effects such as improving flavor and texture. However, overgrowth or unsafe strains can cause spoilage and safety problems.^7^

From count plates, a total of 50 yeasts were isolated, 35 of which came from controlled drying rooms and 15 from natural drying rooms. Takeda et al.^21^ highlights the interaction between the microbiota and the sensory properties of ham, always taking into account that the present yeasts in the products are beneficial and not pathogenic, hence the importance of identifying at the strain level and performing biosafety tests to confirm this.

### 3.2 Genotyping, Identification, and Sequencing of Yeasts

To differentiate the genetic profiles of the isolates (50 from the surfaces of hams and hangers, 35 from controlled and 15 from natural drying rooms), the RAPD-PCR technique was used. Then, PCR-RFLP was applied to different strains to identify each profile. After that, the identification obtained was compared to those found in the sequencing process. Table 3 shows all of the results.

**Table 3:** Classification of the Strains by the PCR-RFLP Profile and DNA Sequencing^b^

	Profile	ITS size (bp)	***Hinf*** I (bp)^a^	***Hae*** III (bp)^a^	***Cfo*** II (bp)^a^	Closet relative specie	Isolates	% confidence
Controlled drying room	I	600	300	400 + 150 + 90	300 + 290 + 50	*Debaryomyces hansenii*	**C1S2-1**, C1H1- 1, C1S2-1, C2S2-1, C2S2-3, C1S3-1, C1S1-1, C1S1-2, C1S1-3, C2S3-1, C2S2-2, C2S2-4, C1H2-5, C1H1-3	92.5%
	II	350	190	360	200 + 190	*Yarrowia lipolytica*	**C1H1- 2**, C1H1-4, C3H1-1, C3H1- 2	98.1%
	III	600	310	150 + 400	290 + 500	*Candida zeylanoides*	**C2H2-2**	97.9%
	IV	800	300 + 120	300 + 210 + 150 + 120	300 + 150	*Saccharomyces cerevisiae*	**C2S2-2**, C2S1- 1, C2S1-2	92.1%
	V	390	200 + 150 + 80	400	200	*Diutina rugosa*	**C1H1-3**, C3H1-4	97.4%
	VII	600	315 + 300 + 290	400 + 290 + 150	250 + 200 + 190	*Debaryomyces castelli*	**C2H2-1**	99.5%
	VIII	390	200 + 400	150 + 290 + 400	200	*Starmerella koui*	C2S1-3, C**2S1- 4**, C2S1-5	97.0%
Natural drying room	I	600	300	400 + 150 + 90	300+ 290+ 50	*Debaryomyces hansenii*	N4I3-2 N4I3-4, N4I3-3, N4I1-4, **N5S2-1**, N5S2- 2	92.5%
	VI	625	220 + 300 + 70	200 + 400	90+ 200+ 300	*Rhodotorula mucilaginosa*	N4I1-1, **N4I1-2**, N4I1-3, N4I3-1, N5S1- 1, N5S1-2, N5I-3	98.9%

a Codes in bold indicate the representative strain chosen for sequencing. **C** (controlled drying room), **N** (natural drying room), **S** (Serrano ham), **I** (Iberian ham), **H** (ham hangers), **A** (air). A number was assigned to each sampling point to complete the code.

b Restriction fragments smaller than 50 bp could not be visualized.

Of the 50 isolates, 9 could not be amplified in the first PCR (RAPD-PCR). The other 41 were grouped into 8 different species. The 34 yeast isolates from the controlled drying rooms were classified into 7 different species: *Debaryomyces hansenii*, *Yarrowia lipolytica*, *Candida zeylanoides*, *Saccharomyces cerevisiae*, *Debaryomyces castelli*, *Diutina rugosa* and *Starmerella koui*. On the other hand, the 7 isolates corresponding to the natural drying rooms were distributed in 2 profiles: *Rhodotorula mucilaginosa* and *D. hansenii*. Whereby, *D. hansenii* is the predominant species and the only one found in both natural and controlled drying rooms.

Regarding the species found, the literature confirms that the genera *Debaryomyces*, *Saccharomyces*, *Candida* and *Yarrowia* are frequently isolated from meat environments.^22,23^ Among them, the predominant yeast species in cured meat products, such as dry-cured ham, is *D. hansenii*, which is in concordance with the results presented (44.0% of the species identified). This specie is characterized by its tolerance to high salt concentrations, which explains its frequent presence in cured products. Moreover, *D. hansenii* has also been associated with the production of volatile compounds that contribute to the characteristic aroma of cured hams.^24,25^ They are mostly aldehydes, ketones, alcohols, esters, and sulfur compounds, contributing significantly to the final characteristic aroma of cured hams.^23^

A slight influence of the unequal number of samples between controlled and natural drying rooms should be taken into consideration in the analysis of the distribution of *D. hansenii*. From a broader methodological perspective, ecological studies have shown that when sampling is uneven or based only on presence data, the resulting estimates of species distribution can be biased.^26^ Nevertheless, as mentioned before, the dominance of *D. hansenii* in dry-cured meat related environments has been previously proved, although its abundance can vary depending on product type and production conditions.^9,27^ While this imbalance in the number of samples between drying rooms could potentially influence the apparent prevalence of *D. hansenii*, the consistent detection patterns observed across both room types suggest that the general distribution trends reported here remain robust.

The second most prevalent species was *Y. lipolytica* (19.5%). It is responsible for lipolysis processes in cured meat products during the maturation stage, releasing free fatty acids in large quantities and contributing to the formation of flavors and aromas.^23,28^ Other yeasts belonging to the species *S. koui*, *D. rugosa* and *R. mucilaginosa* have been identified in the samples, although they are not of major described relevance in meat products. These yeasts are found occasionally and are mainly associated with the presence of surfaces and equipment where meat is handled.^29^

### 3.3 Biodiversity of Meat Environments

To evaluate the biodiversity of yeast species in the different drying rooms, Simpson’s index was calculated, taking into account the number of isolates of each species and the total number of isolates in each type of drying room. The results showed a higher biodiversity in the controlled drying rooms, with a Simpson’s index of 0.73, compared to the natural ones, which had an index of 0.65. These differences in biodiversity may be due to the fact that more samples were taken in the controlled (*n* = 19) compared to the natural ones (*n* = 8). In addition, the controlled drying rooms contained a greater number of hams for production and there was a greater variety in the origins and provenances of the hams. Those factors could have contributed to the diversity of ecological niches, allowing the establishment of a greater number of yeast species.

On the other hand, the percentage of genetic variability was used to study the biodiversity of strains within the same species (Figure 2).

**Figure 2:**
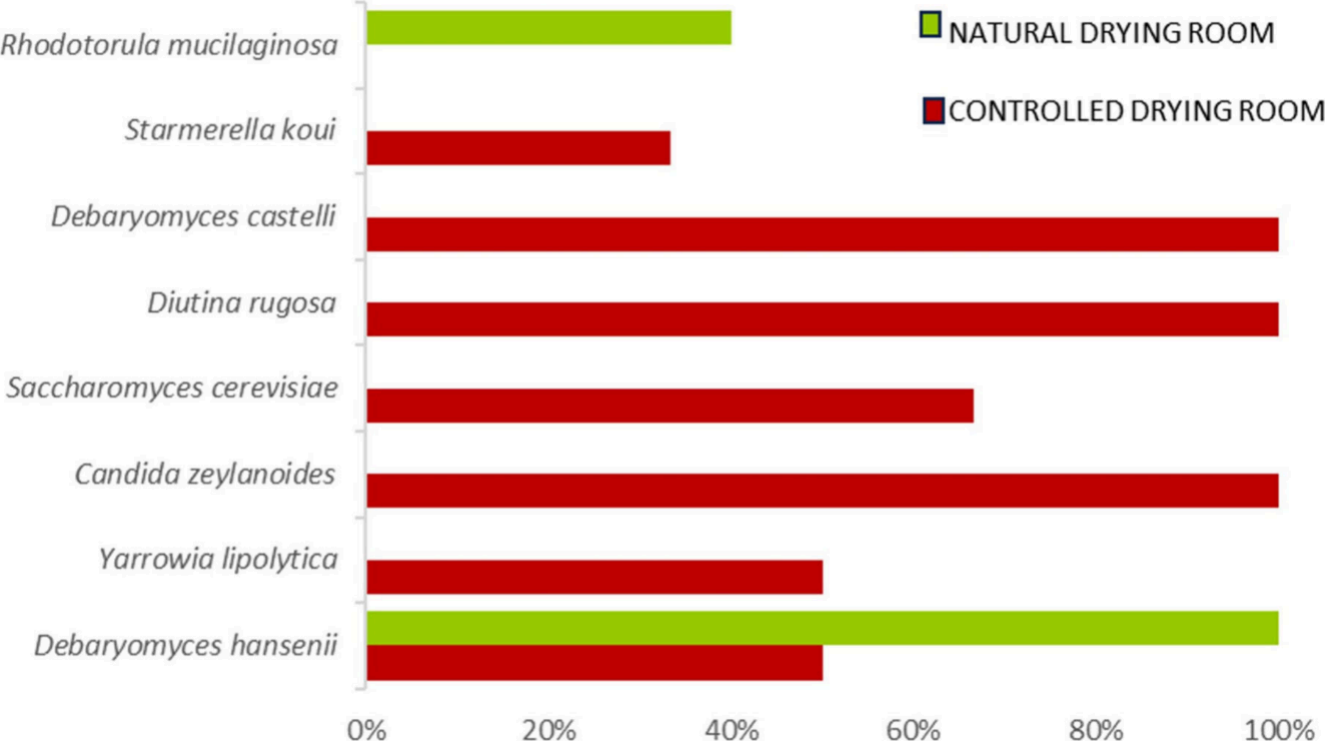
Percentages of genetic variability of strains isolated from dry-cured ham.

This analysis revealed that hams from controlled drying rooms exhibited 100% genetic variability in *D. castelli*, *D. rugosa*, and *C. zeylanoides*, which may have positive implications for the adaptability and functionality of these yeasts in fermentation and production processes. In contrast, dry-cured hams from natural drying rooms showed that *D. hansenii* was the species with the highest percentage of genetic variability, indicating a significant level of genetic diversity in this environment.

However, *S. koui* displayed lower genetic variability in the controlled drying rooms (33.3%) compared to the other species. This was followed by *Y. lipolytica* and *D. hansenii*, both with 50% genetic variability. In natural drying rooms, *R. mucilaginosa* also presented a genetic variability of 50%, suggesting that although there is remarkable diversity, it did not reach the levels observed in strains from controlled drying rooms. These observations suggest that highly controlled environments can exert selective pressures that differentially affect yeast genetic variability; while some species exhibited high variability, others, such as *S. koui*, appeared less variable under uniform conditions. This aligns with Deák’s^30^ study, in which it is emphasized that environmental factors (e.g., temperature, humidity, salt concentration, etc.) shape yeast populations by favoring certain genotypes or contributing to the heterogeneity of specific species. Additionally, previous molecular studies on Iberian dry-cured hams from natural drying rooms, revealed high intraspecific variability within dominant yeast species such as *D. hansenii* and *C. zeylanoides*, and even identified biotypes associated with geographical areas.^9,31^ Such results highlight that yeast populations in dry-cured ham environments are not homogeneous but rather are influenced by the ripening conditions.

Several studies have proven the biotechnological interest of yeasts isolated from cured ham. Virgili et al.^32^ evaluated the ability of these yeasts to control the production of ochratoxin A in meat models, identifying strains such as *D. hansenii* with significant inhibitory activity. On the other hand, previous research has shown that yeasts isolated from Iberian cured ham have a high potential for the synthesis of volatile compounds responsible for the development of the aromatic profile, which is advantageous for the production of ingredients with desirable sensory characteristics.^33^ Furthermore, it has been shown that certain strains have probiotic properties, such as the ability to produce antioxidant peptides.^34^ These results indicate that cured ham yeasts are not only useful in the food industry but also could be candidates for obtaining single-cell microbial biomass due to their functional properties.

### 3.4 Screening and Characterization of Yeasts

#### 3.4.1 Growth Capacity

To identify the yeasts with the best growth characteristics, a growth kinetic analysis was carried out. This study provided data on the following kinetic parameters: the lag phase (λ), the maximum growth rate (μ max), the generation time (*G*), and the maximum optical density (ODmax). The results are detailed in Table 4, providing a solid basis for the selection of the most promising yeast strains in terms of growth and productivity.

**Table 4:** Values of the Kinetic Parameters of the Yeasts Studied^a^

		**Kinetic parameters**			
**Strain Number (Specie)**		**λ** (h)	**μ max** (h^–1^)	***G*** (h)	**ODmax**
**C1S2-1**	*D. hansenii*	7.71 ± 0.22^f,g^	0.08 ± 0.00^d,e,f^	3.82 ± 0.14^b^	1.44 ± 0.23^f,g^
**C2S2-2**	*S. cerevisiae*	4.50 ± 0.08^b^	0.13 ± 0.00^g^	2.57 ± 0.29^a^	1.39 ± 0.34^e,f,g^
**C1H1-1**	*D. hansenii*	7.32 ± 0.28^f^	0.14 ± 0.00^g,h^	2.10 ± 0.09^a^	1.45 ± 0.34^f,g^
**N5S2-1**		16.60 ± 0.50^j^	0.04 ± 0.00^a,b^	7.72 ± 0.03^f,g^	1.41 ± 0.08^f,g^
**C2S2-3**		18.47 ± 0.45^l^	0.18 ± 0.00^i^	2.59 ± 0.11^a^	0.47 ± 0.02^a^
**C2S2-1**		6.59 ± 0.11^e^	0.13 ± 0.02^g^	2.46 ± 0.14^a^	1.91 ± 0.11^i^
**N5S2- 2**		7.51 ± 0.07^f,g^	0.09 ± 0.00^d,e,f^	3.50 ± 0.06^b^	1.78 ± 0.12^h^
**C2H2-2**	*C. zeylanoides*	17.28 ± 0.35^k^	0.06 ± 0.00^c^	7.81 ± 0.15^f,g^	1.57 ± 0.12^g,h^
**C1H1-2**	*Y. lipolytica*	3.14 ± 0.10^a^	0.14 ± 0.02^g,h^	3.64 ± 0.06^b^	1.08 ± 0.07^c,d,e^
**C1H1-3**	*D. rugosa*	7.32 ± 0.26^f^	0.03 ± 0.00^a^	8.11 ± 0.13^g^	0.86 ± 0.11^b,c^
**C1S1-1**	*D. hansenii*	3.20 ± 0.15^a^	0.08 ± 0.00^d,e^	23.44 ± 0.39^i^	1.45 ± 0.11^f,g^
**C2S1-1**	*S. cerevisiae*	5.19 ± 0.17^c^	0.09 ± 0.00^e,f^	2.35 ± 0.30^a^	1.59 ± 0.42^g,h^
**C2S1-4**	*S. koui*	14.39 ± 0.53^i^	0.13 ± 0.02^g^	2.18 ± 0.13^a^	0.96 ± 0.01^c,d^
**C1H2-5**	*D. hansenii*	14.69 ± 0.10^i^	0.04 ± 0.01^a,b^	7.56 ± 0.48^f^	1.19 ± 0.14^d,e,f^
**C2S2-4**		6.40 ± 0.33^d,e^	0.17 ± 0.02^i^	2.49 ± 0.02^a^	1.79 ± 0.19^h,i^
**C2H2-1**	*D. castelli*	7.26 ± 0.17^f^	0.09 ± 0.00^e,f^	3.48 ± 0.10^b^	2.04 ± 0.05^i^
**N4I1-2**	*R. mucilaginosa*	6.46 ± 0.34^d,e^	0.15 ± 0.01^h^	2.15 ± 0.19^a^	1.37 ± 0.10^e,f,g^
**C1H1-4**	*Y. lipolytica*	12.20 ± 0.19^h^	0.02 ± 0.00^a^	17.56 ± 0.40^h^	0.62 ± 0.06^a,b^
**C3H1-4**	*D. rugosa*	6.02 ± 0.11^d^	0.06 ± 0.01^c^	5.51 ± 0.48^d^	0.80 ± 0.09^b,c^
**N5S1-1**	*R. mucilaginosa*	7.92 ± 0.40^g^	0.07 ± 0.00^d^	4.64 ± 0.38^c^	0.83 ± 0.03^b,c^
**C3H1-1**	*Y. lipolytica*	3.49 ± 0.34^a^	0.05 ± 0.00^b,c^	30.84 ± 0.50^j^	1.28 ± 0.24^e,f,g^
**C1H1-3**	*D. hansenii*	5.47 ± 0.44^c^	0.08 ± 0.01^d,e^	6.16 ± 0.10^e^	0.90 ± 0.06^b,c,d^
**Mean ± SD**		8.60 ± 4.67	0.09 ± 0.06	6.94 ± 7.38	1.28 ± 0.44
**Mode**		-	0.02	7.70	-
**Median**		7.17	0.08	3.71	1.28
**Maximum value**		18.88	0.19	31.17	2.10
**Minimum value**		3.02	0.02	2.00	0.46

a **C** (controlled drying rooms), **N** (natural drying rooms), **S** (Serrano ham), **I** (Iberian ham), **H** (ham hangers), **A** (air). A number was assigned to each sampling point to complete the code. Different superscripts indicate significant differences between the mean values in each column (*p* < 0.05).

The λ values, which indicate the time taken for the yeast to adapt to the conditions of the medium, ranged between 3.02 and 18.88 h for *Y. lipolytica* (C1H1-2) and *D. hansenii* (C2S2-3) respectively. Significant differences (*F* = 770.753) were shown between the samples, differentiating 11 groups. Regarding the μmax values, which reflect the rate at which the yeast population multiplies in its exponential phase, 8 different groups were observed (*F* = 85.027) being 0.09 ± 0.06 h^–1^ the μmax mean. The fastest yeast was *D. hansenii* (C2S2-3 and C2S2-4). *G*, which measures the time required for the population to double, showed large significant differences (*F* = 2426.381) among the yeasts, ranging from 2.00 to 31.17 h, belonging to R. *mucilaginosa* (N4I1-2) and *Y. lipolytica* (C3H1-1) respectively. Fifty percent of the strains studied had a *G* < 3.71 h. Finally, for ODmax, that represents the maximum cell concentration reached during growth, the mean value was 1.28 ± 0.44, which was exceeded by 50% of the strains. Nine groups were differentiated with significant differences between the samples (*F* = 17.601). In contrast, some yeasts showed very low values, such as *D. hansenii* (C2S2-3), *D. rugosa* (C1H1-3, C3H1-4) and *Y. lipolytica* (C1H1-4). This behavior was also reflected in the rest of the parameters: long λ, low μmax, and high *G* value.

Yeast selection was carried out considering two key parameters: OD max and λ. This approach ensured that the chosen strains showed efficient growth, short adaptation time, and high productivity, as mentioned in the study by Chacón-Villalobos and Viveros-Lizondo et al.^17,19^ Limits for yeast selection were set at <6 h for λ and at >1 for ODmax. Strains that did not meet these conditions were discarded from the selection process, thus ensuring that only the most promising yeasts advanced to subsequent stages of the study. In that case, 5 strains meet the criteria established above, representing 22.72% of the yeasts present in the study, and these belong to the species *S. cerevisiae* (C2S2-2), *Y. lipolytica* (C1H1-2), *D. hansenii* (C1S1-1), *S. cerevisiae* (C2S1- 1) and *Y. lipolytica* (C3H1-1).

#### 3.4.2 Protein Production

To determine the total protein content produced by the selected yeasts, their dry biomass was subjected to ultrasound treatment (50W for 20 min) to facilitate protein release. Then, the protein content of each strain was quantified using the Bradford method (Figure 3).

**Figure 3:**
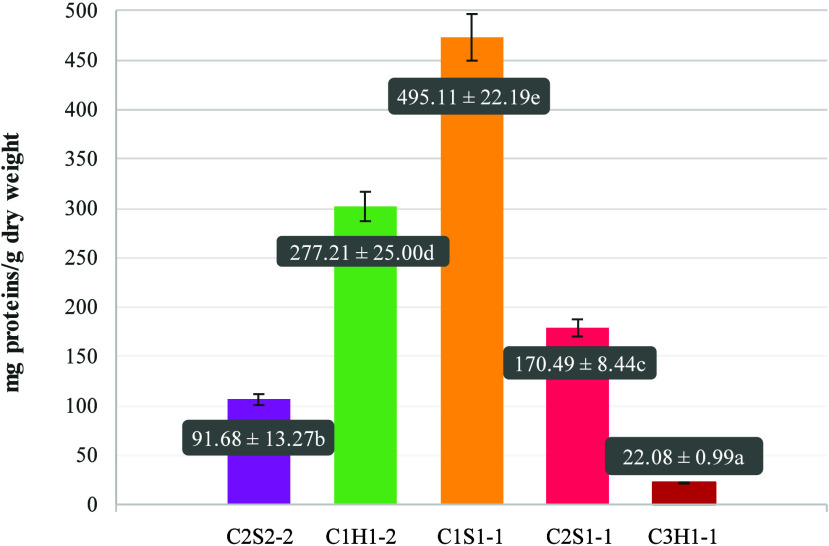
Protein content of yeast after ultrasound treatment (mg protein/g dry weight of yeast). Different letters in the values indicate significant differences between the protein contents (*p* < 0.05).

The strain with the highest protein production was C1S1-1 (*D. hansenii*), with 495.11 ± 22.19 mg of protein/g of dry weight. In the case of the two strains belonging to *Y. lipolytica*, great variability was observed. Strain C1H1-2 obtained a value of 277.21 ± 25.00 mg of protein/g of dry weight, while C3H1-1 had a much lower protein content of only 22.08 ± 0.99 mg of protein/g of dry weight. Finally, *S. cerevisiae* strains C2S2-2 and C2S1-1 presented intermediate values of 91.68 ± 13.27 and 170.49 ± 8.44 mg of protein/g of dry weight, respectively.

Furthermore, significant differences were identified among the 5 samples, which was determined by ANOVA statistical treatment, with a significance level of 0.05 and a value of *F* = 375.174. Subsequently, Duncan’s test revealed that each strain was found in a different group regarding its protein content. Results indicate that the mycoprotein content and its liberation are highly strain-dependent characteristics.

Following the criterion for selecting yeasts indicated by Viveros-Lizondo et al.,^19^ the most promising strains were those with more than 50 mg protein/g dry weight. This value was exceeded by all of the strains evaluated, except for C3H1-1 (*Y. lipolytica*), which presented a content of 22.08 ± 0.99 mg of protein/g of dry weight and therefore was discarded for the next assays.

As demonstrated in this study, yeasts are considered to be a good source of protein; their content can vary significantly depending on the species and even the study strain. Growth conditions (nutrient availability, temperature, and pH) and sample treatment during drying must also be taken into account.^35^ Fleet^36^ estimated that *S. cerevisiae* contains close to 50% of protein on a dry weight basis. Pobiega et al.^37^ support that the amount of protein in dry weight that can be contributed by *S. cerevisiae* and *Y. lipolytica* can be up to 40% and 50%, respectively; these genera are of interest due to the ability to produce proteolytic enzymes, which contribute to the breakdown of proteins, thus improving the sensory characteristics of cured ham. However, other species, such as *C. utiliz* and *K. marxianus*, are known for their high protein content and can reach up to 55% dry weight under optimal conditions.

It is essential to know the optimal growth conditions to produce single-cell protein, as there is a large difference in the initial protein content. For example, Liu et al.^38^ studied the growth of *D. hansenii* for this purpose, considering variations in pH and temperature. This species is of interest in the meat industry, particularly in ham drying rooms, as it is a halotolerant yeast. The conditions established for maximizing biomass and, therefore, protein content have been a neutral pH and a temperature of 28 °C. Other studies have indicated that with optimal conditions for *C. utiliz* and *K. marxianus*, they can reach up to 55% protein content in dry weight.^36^

#### 3.4.3 Metabolism Inactivation

In order to guarantee the inhibition of yeasts after ultrasound treatment, the viability of each strain was assessed by its inoculation on YM plates after the treatment. Once incubated, strain C2S2-2 (*S. cerevisiae*) showed normal growth, indicating that the ultrasound treatment did not have a negative impact on its viability. On the other hand, strain C1H1-2 (*Y. lipolytica*) experienced a significant decrease in its growth capacity but was not completely inhibited, which was interpreted as partial inhibition or attenuation of metabolisms. In contrast, strains C1S1-1 (*D. hansenii*) and C2S1- 1 (*S. cerevisiae*) were totally inhibited, and they were not able to grow after the ultrasound process.

This test was carried out since total metabolism inactivation would be essential to avoid undesired microbiological modifications and alterations in the sensorial characteristics (smell, taste, and texture) in the product where yeasts will be added as an ingredient for reducing animal protein content. For example, the production of organic alcohols and acids can alter the taste, making it bitter or sour, and contributes to the development of unpleasant odors, such as fermented or decomposing aromas. In addition, it can lead to changes in the texture of the product, resulting in a more viscous or sticky texture. Therefore, it is essential to introduce inactivated yeasts to prevent their growth during product development or storage to ensure the stability, safety, and acceptability of the final products.^39,40^

Therefore, considering all results, the unique selected yeasts were C1S1-1 (*D. hansenii*) and C2S1-1 (*S. cerevisiae*), which indicated that only 9.09% of them passed all of the selection criteria proposed in this study.
